# External Validation of the Acute Kidney Injury Risk Prediction Score for Critically Ill Surgical Patients Who Underwent Major Non-Cardiothoracic Surgery

**DOI:** 10.3390/healthcare9020209

**Published:** 2021-02-15

**Authors:** Konlawij Trongtrakul, Jayanton Patumanond, Piyarat Phairatwet, Chaiwut Sawawiboon, Anusang Chitsomkasem, Sathit Kurathong, Surasee Prommoon, Thananda Trakarnvanich, Phichayut Phinyo

**Affiliations:** 1 Department of Internal Medicine, Faculty of Medicine, Chiang Mai University, Chiang Mai 50200, Thailand; konlawij@live.com; 2 Department of Internal Medicine, Faculty of Medicine Vajira Hospital, Navamindradhiraj University, Bangkok 10300, Thailand; pphairatwet@gmail.com (P.P.); hlexxxx@gmail.com (C.S.); anusang@nmu.ac.th (A.C.); sathit@nmu.ac.th (S.K.); surazee@nmu.ac.th (S.P.); thananda@nmu.ac.th (T.T.); 3 Department of Clinical Epidemiology, Faculty of Medicine, Thammasat University, Pathum Thani 12120, Thailand; 4 Center for Clinical Epidemiology and Clinical Statistics, Faculty of Medicine, Chiang Mai University, Chiang Mai 50200, Thailand; jpatumanond@gmail.com; 5 Department of Family Medicine, Faculty of Medicine, Chiang Mai University, Chiang Mai 50200, Thailand; 6 Musculoskeletal Science and Translational Research (MSTR) Cluster, Chiang Mai University, Chiang Mai 50200, Thailand

**Keywords:** acute kidney injury, prediction, critical care, surgery, validation study

## Abstract

Background: Acute kidney injury (AKI) is a common complication encountered in an intensive care unit (ICU). In 2020, the AKI prediction score was developed specifically for critically ill surgical patients who underwent major non-cardiothoracic surgeries. This study aimed to externally validate the AKI prediction score in terms of performance and clinical utility. Methods: External validation was carried out in a prospective cohort of patients admitted to the ICU of the Faculty of Medicine Vajira Hospital between September 2014 and September 2015. The endpoint was AKI within seven days following ICU admission. Discriminative ability was based on the area under the receiver operating characteristic curves (AuROC). Calibration and clinical usefulness were evaluated. Results: A total of 201 patients were included in the analysis. AKI occurred in 37 (18.4%) patients. The discriminative ability dropped from good in the derivation cohort, to acceptable in the validation cohort (0.839 (95%CI 0.825–0.852) vs. 0.745 (95%CI 0.652–0.838)). No evidence of lack-of-fit was identified (*p* = 0.754). The score had potential clinical usefulness across the range of threshold probability from 10 to 50%. Conclusions: The AKI prediction score showed an acceptable discriminative performance and calibration with potential clinical usefulness for predicting AKI risk in surgical patients who underwent major non-cardiothoracic surgery.

## 1. Introduction

Acute kidney injury (AKI) has been increasingly reported globally [1,2]. The prevalence of AKI was reported to be much greater in patients who were admitted to an intensive care unit (ICU) [3,4,5,6], and was associated with significant morbidities [7,8] and mortalities [4,6,9]. Critically ill surgical patients are particularly vulnerable to AKI [6,9]. The stress induced by surgical procedures and anesthetic drugs may cause acute deterioration of kidney function [10]. Many factors besides fluid depletion and consequences of operations presumably play an important role in the pathogenesis of AKI in surgical patients during peri-operative events, such as neurohormonal compensatory response to anesthetic agents inducing vasodilation [11], peri-operative blood loss [12], and intra-operative hypotension [13]. As the etiologic mechanism of AKI in surgical patients is markedly different from those of the medical patients, specific approaches and management of AKI are needed.

Early detection of AKI is postulated to be crucial during early AKI management [2,14]. A decision must be made whether the initiation of early aggressive intervention to prevent the deterioration of kidney function is required. Several clinical prediction tools for prompt AKI detection were developed to aid physicians in early AKI identification and risk stratification for further management [2]. However, most AKI prediction scores for surgical patients were derived from a cohort of patients who either underwent cardiothoracic surgery [15,16], orthopedic surgery [17], or liver resection and liver transplantation surgery [18,19]. AKI prediction scores developed specifically for critically ill surgical patients who underwent other major non-cardiothoracic surgeries are limitedly reported [20] and seldomly validated [20,21,22].

In 2020, the clinical score for predicting AKI in surgical patients who underwent major non-cardiothoracic surgery and were admitted to the ICU was developed from a large prospective multi-center observational cohort in Thailand [23]. To the best of our knowledge, this AKI prediction score was the only score that was specifically developed for this specific domain of surgical patients (i.e., major non-cardiothoracic surgery, other than orthopedic surgery, liver resection, or liver transplantation). The score exhibited excellent discriminative performance with good calibration and a minimal degree of optimism. However, an external validation study is required to provide evidence on the score performance outside the derivation samples prior to its clinical implementation [24]. This study aimed to validate the recently developed AKI prediction score in an external dataset in terms of discriminative ability, calibration, and clinical usefulness.

## 2. Materials and Methods

An external validation study of a recently developed AKI prediction score was performed. Two patient cohorts were used in the analysis of this study, which were the derivation cohort of the AKI prediction score and the external validation cohort. This study was conducted and reported in compliance with the Transparent reporting of a multivariable prediction model for individual prognosis or diagnosis (TRIPOD) [25]. 

The AKI prediction score was developed based on the multi-center, university-based, observational cohort study in Thailand, named THAI-SICU Study [26]. Between April 2010 and January 2013, 4652 critically ill surgical patients were included in the cohort. A screening criterion was used to identify the proper study domain for prediction as follows: adult patients (age equal to 18 and over) who underwent a major non-cardiothoracic operation and were admitted to the ICU for more than 24 hours. A total of 3474 critically ill surgical patients were used during the AKI prediction score derivation [23]. The incidence of AKI within seven days was 9.6%. In the development study, AKI was defined according to the Kidney Disease: Improving Global Outcomes (KDIGO) 2012-serum creatinine criteria [27].

The AKI prediction score incorporates pre-operative, peri-operative, and post-operative characteristics for the prediction of AKI, which were the patient age, emergency of surgery, peri-operative blood loss, peri-operative urine output, presence of sepsis, and the Sequential Organ Failure Assessment (SOFA) non-renal score at ICU admission. The summation of the score was 16.5. The score categorizes patients into four risk groups as follows: low risk (0.0–2.5), moderate risk (3.0–8.5), high risk (9.0–11.5), and very high risk (12.0–16.5). The apparent discriminative ability of the score based on the area under the receiver operating characteristic curve (AuROC) was 0.839 (95%CI 0.825–0.852). The score also showed acceptable calibration, according to Hosmer–Lemeshow (HL) statistic (*p*-value = 0.302) [23].

The validation cohort was based on the prospective observational study of critically ill surgical patients admitted during September 2014 and September 2015 at the Faculty of Medicine Vajira Hospital, Navamindradhiraj University, Bangkok, Thailand [6]. As our center is one of the centers that participated in the THAI-SICU study, we only selected patient records that were not included in the derivation cohort and were admitted at least three years apart from the data in the derivation cohort.

In the validation cohort, all included patients were followed for a duration of 28 days after their ICU admission. The study primarily aimed to determine the prevalence of AKI and its impacts on mortality, by using KDIGO-2012 serum creatinine and modified urine output as diagnostic criteria. Originally, 400 cases were enrolled. The prevalence of AKI was 47.3% (189 patients). For external validation, we selected only adult patients admitted to the surgical ICU because of high-risk operations (decision authorization based on surgeon and anesthesiologist preferences, without any manipulations by the researchers). ICU admissions due to acute medical reasons rather than immediate post-operation (e.g., acute myocardial infarction, congestive heart failure, and stroke) were excluded. Patient records with incomplete data on predictors and outcomes were also excluded.

The endpoint for prediction was the occurrence of AKI, which was defined according to the KDIGO-2012 serum creatinine criteria only (i.e., an increase in serum creatinine of at least 0.3 mg/dL within 48 hours, or an increase in serum creatinine more than 1.5 times the baseline serum creatinine) [27]. The point of prediction was within the first day of ICU admission, or as soon as all predictors were available. The predicted endpoint was the occurrence of AKI within seven days after ICU admission.

Baseline characteristics of the patients were collected, including: (1) the pre-operative demographics data (age, gender, body weight, and body mass index), (2) the pre-existing comorbidities (diabetes mellitus, hypertension, cardiovascular diseases, respiratory diseases, chronic kidney disease, malignancy, and others), (3) the information provided from peri-operative period (the American Society of Anesthesiologists (ASA) classification, emergency operation, type and duration of operation, peri-operative blood loss, fluid balance, and urine output), (4) the severity of illness upon ICU admissions (Acute Physiology and Chronic Health Evaluation-II (APACHE-II) score, the Sequential Organ Failure Assessment (SOFA) score, and SOFA non-renal score), (5) the presence of sepsis at the ICU admission, (6) the initial laboratory investigations upon ICU admissions (hemoglobin, albumin, blood sugar, PF ratio, chest imaging, electrocardiography, and serum creatinine and its reference level), (7) the endpoints of interest from each cohort (AKI within seven days of the ICU admission, ICU mortality, day-28 mortality, ICU length of stay, and hospital length of stay), and (8) calculated AKI prediction score.

Continuous data were described with mean and standard deviation (SD). Categorical data were reported as frequency and percentages. Standardized difference (STD) was used to compare differences in baseline characteristics between the derivation and validation cohorts. An STD value beyond the ranges of −0.200 to 0.200 was considered as a significant difference [28]. Stata 16 (StataCorp, College Station, TX, USA) was used for all statistical analyses.

We assess the predictive performance of the AKI prediction score in terms of discrimination and calibration. Discrimination determines how well the prediction score can differentiate patients with the outcome (AKI) from patients without (non-AKI). To assess discrimination, AuROC was used [29]. Calibration indicates the agreement between the score predicted probabilities and the observed proportion of outcome occurrence. The score calibration was statistically assessed by the HL goodness-of-fit test and graphically plotted by the predicted probability of AKI and the observed risk of AKI for each score. Positive predictive values (PPV) were estimated for each score category to represent the predicted risk of AKI. We also used the STD to compare the AuROC and the PPV between the derivation and the validation cohort.

We also measured the clinical usefulness of the AKI prediction score as a directive tool for suggesting an early aggressive treatment for AKI with the use of decision curve analysis (DCA) to estimate the net benefit (NB) [30]. DCA depicts the NB gained from applying the prediction score in practice, based on the threshold probability compared to two default strategies, including providing early aggressive treatment to all (treat all) and not providing early aggressive treatment to any patients (treat none). The NB of the prediction score should be higher than those two default strategies; otherwise, no additional benefit is gained. We also calculated the NB of the AKI prediction score based on a weighted sum of true-positive minus false-positive classification according to the threshold probability. The formula of NB was calculated from ((TP−w x FP)/N), where TP was the number of true-positive classifications, FP was the number of false-positive classifications, w was the weight that equaled the odds of the threshold probability, and N was the total sample size [31]. A step-by-step guide for interpretation of decision-curve analysis was recently published to help readers understand the DCA concept [32].

Additional analysis was performed to examine the differences in the effect of all the predictors within the AKI prediction score on the occurrence of AKI between the derivation cohort and the external validation cohort.

## 3. Results

**Figure 1:**
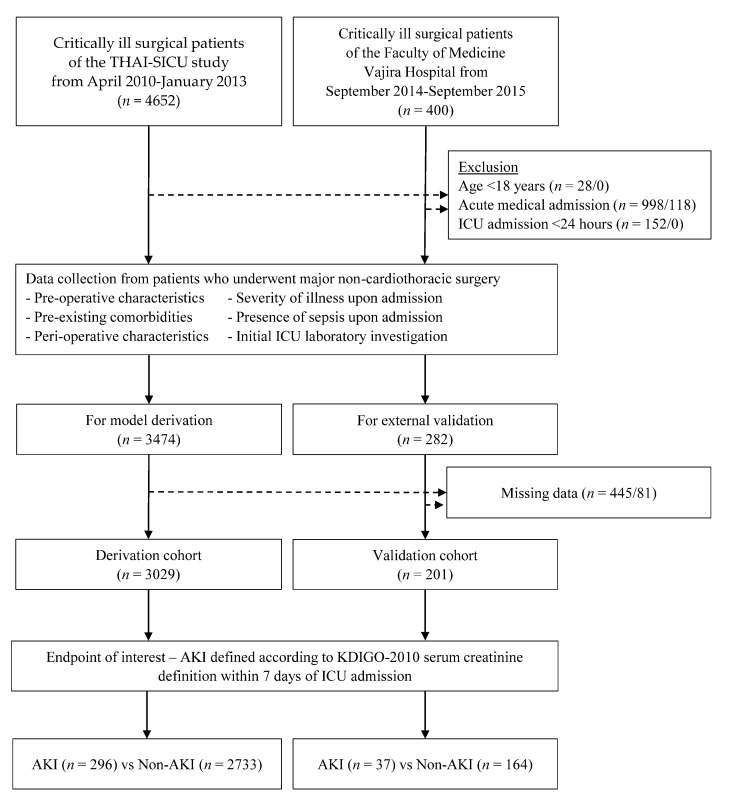
Flow chart of the study patients in the derivation and the validation cohort.

Four hundred patients in the validation cohort were screened for eligibility. One hundred and eighteen patients were excluded due to acute medical reasons for the ICU admission not related to surgical operation, and 81 patients were excluded due to incomplete data. The remaining 201 patients were included in the score validation. Of the patients, 37 (18.4%) experienced AKI within seven days following their ICU admission (Figure 1). 

Table 1 compares the patient profiles between the derivation and the validation cohort. Most of the patients’ demographics, pre-existing comorbidities, and type of surgical operation were similar. However, a larger proportion of patients with chronic kidney disease, malignancy, and others were identified in the validation cohort. For peri-operative characteristics, ASA physical status (STD = 0.259), orthopedic surgery (STD = 0.215), and peri-operative fluid balance (STD = 0.263) were significantly different. Overall, the patients in the validation cohort were clinically more severe than the patients in the derivation cohort, according to the severity scores (APACHE-II (STD = −0.470), SOFA (STD = −0.371), and SOFA non-renal scores (STD = −0.524)). Although the proportion of patients with sepsis was not significantly higher in the validation cohort than in the derivation cohort, the patients had lower hemoglobin (STD = −0.351) and serum albumin levels (STD = −0.322). The incidence of AKI was almost doubled in the validation cohort (9.8% vs. 18.4%, STD = 0.198). A significantly longer ICU length of stay was also identified in the validation cohort (STD = −0.341).

**Figure 2:**
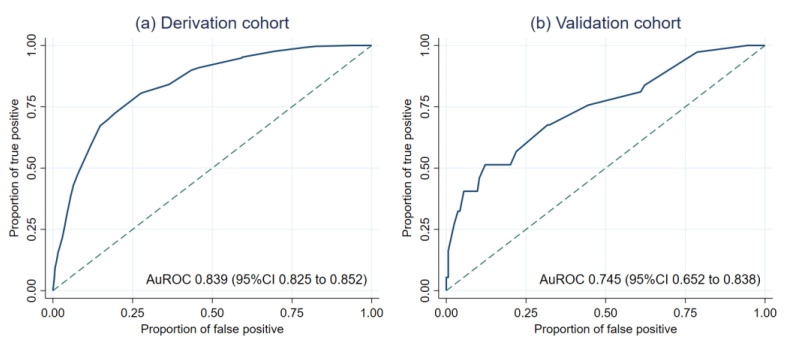
Discriminative ability of the AKI prediction score from the derivation cohort (**a**) and validation cohort (**b**).

**Figure 3:**
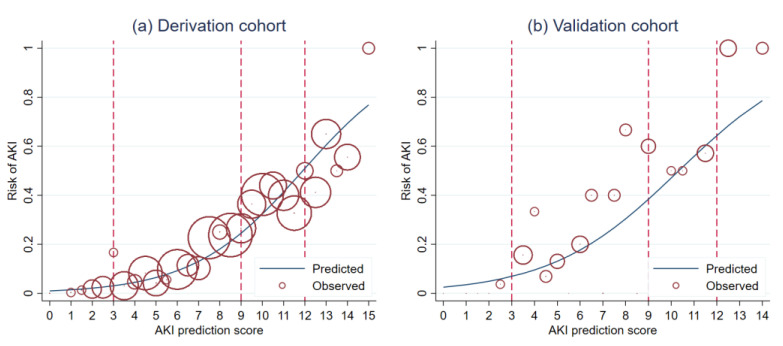
Calibration plots illustrating score predicted risk (solid line) and observed risk (circles) in the derivation cohort (**a**) and validation cohort (**b**).

The average AKI prediction score between the derivation and the validation cohorts were significantly different (8.5 ± 3.2 vs. 7.8 ± 3.5 (STD = 0.216) for patients with AKI, and 4.1 ± 2.9 vs. 4.9 ± 2.3 (STD = −0.279) for patients without AKI, respectively). The detailed description of the performance of the AKI prediction score during the derivation was reported in our previous work [23]. In terms of discriminative ability, the AKI prediction score showed significantly higher discriminative ability in the derivation cohort (Figure 2a) than in the validation cohort (STD = 0.245) (Figure 2b). However, no evidence of lack-of-fit had been detected in both cohorts (HL-statistic *p*-value 0.302 and 0.754, respectively). The calibration of the AKI prediction score in both cohorts were visualized in the calibration plots (Figure 3a,b). Visually, the calibration of the score seemed to be better in the derivation cohort than in the validation cohort.

The AKI prediction score categorized patients into four risk groups: low risk, moderate risk, high risk, and very high risk. The PPVs increased concordantly with the higher risk category in both cohorts (Table 2). The PPVs were comparable between the development and the validation cohort in low to high-risk groups; however, the PPV was significantly lower in the derivation cohort than in the validation cohort in a very high risk group (STD = 0.375). The predictors and their coefficients for AKI prediction from both cohorts are reported in the Supplementary Material Table S1.

**Figure 4:**
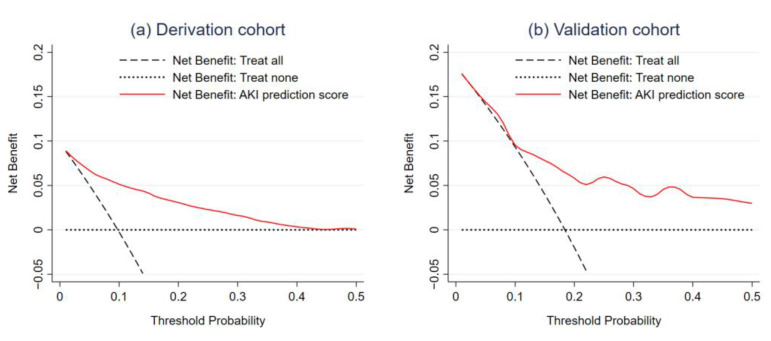
Decision curves visualizing the net benefit for two default strategies, and the AKI prediction score in the derivation cohort (**a**) and validation cohort (**b**).

According to the decision curve, the AKI prediction score demonstrated potential clinical utility in the derivation cohort. The NB of the score was higher than the two default treatment strategies across the range of threshold probability from 5% to 50% (Figure 4a). The score could guide clinicians to provide early aggressive treatment to potential patients with AKI (True Positive), which would reduce the number of overtreatments in patients without AKI (False Positive). For instance, given the threshold probability was 10%, the NB of applying the AKI prediction score was 5.1% compared to −0.3% and 0.0% for treat all and treat none strategies, respectively (Table 3). In other words, an additional 5 per 100 patients were predicted accurately by the AKI prediction score. As the threshold probability increased, the NB of the AKI prediction score decreased. However, compared to a strategy to treat all patients to prevent AKI, applying the AKI prediction score in practice could reduce the number of overly aggressive treatments up to 30 to 80 per 100 patients admitted to the ICU (Table 3).

In the validation cohort, the AKI prediction score did not show potential clinical utility until after the threshold probability of 10% (Figure 4b). At the threshold probability of 5% and 10%, a few additional patients were determined to be in need of early aggressive treatment when compared to the strategy to treat all patients (Table 3), and overtreatments could be avoided in only up to 6 per 100 patients. Only after the threshold probability went beyond 15% was the potential utility of the AKI prediction score shown. About 22 to 67 per 100 patients could avoid unnecessary early aggressive intervention to prevent AKI.

In the additional analysis, the differences in the effect of each predictor within the AKI prediction score on the occurrence of AKI between the derivation and the external validation cohort are presented in Supplementary Table S1.

## 4. Discussion

In this study, we validated the performance of the AKI prediction score in determining the occurrence of AKI at seven days after ICU admission in an external dataset. The discriminative ability of the score in terms of AuROC slightly dropped compared to that of the derivation cohort. Although the HL-statistics were insignificance, the calibration of the score in the validation cohort was undeniably poorer than in the derivation cohort. Regardless of discriminative ability and calibration, the AKI prediction score was demonstrated to be potentially useful for guiding early aggressive intervention for critically ill surgical patients who underwent major non-cardiothoracic surgery in both the derivation and the validation cohort.

The occurrence of AKI has negative impacts on the overall condition of the surgical patients admitted to the ICU [4,6,9]. Using AKI prediction scores in practice is recommended as an option for guiding an early AKI management plan [2,14]. The clinical usefulness of AKI prediction scores lies within the ability to provide an accurate prediction of patients who are likely to have AKI postoperatively so that early aggressive intervention to prevent AKI could be timely initiated (i.e., administration of aggressive fluid resuscitation, continuous central venous pressure monitoring, recording hourly urine output, and assessment of fluid responsiveness). These interventions might impose a significant burden on health care workers, especially in settings with limited resources, and it may cause additional risk to the patients in some circumstances. Ideally, aggressive interventions should only be given to high-risk patients. Unnecessary overtreatment in patients with low risk should be avoided.

In the original study of the AKI prediction score, patients with KDIGO AKI stage-I were included as AKI, which minimizes the differences between patients with and without AKI. For this reason, the discriminative ability of the AKI prediction score was only acceptable. Indeed, we still have limited therapeutic choices for AKI, specifically for KDIGO AKI stage-I. However, KDIGO AKI stage-I and non-AKI patients have to be managed differently in practice. For non-AKI patients, there is no need for close monitoring of urine output or frequent blood sampling for serum creatinine, which is in contrast to patients with KDIGO AKI stage-I. Applying the strategy to monitor all patients or take frequent blood samples might be burdensome in settings with limited ICU resources. In this situation, we included patients with KDIGO AKI stage-I as our clinical endpoint of interest, as we believe that these patients should be managed differently from patients without AKI and still should be considered high-risk patients. Moreover, patients with KDIGO AKI stage-I may progress to a higher AKI stage if not timely detected and appropriately managed.

According to the DCA, it is apparent that the application of the AKI prediction score would increase the proportion of net true positive AKI cases and decrease the number of unnecessary overtreatments, compared to the two default treatment strategies. The NB of our AKI prediction score increased across the range of threshold probability of 10 to 50% in both the derivation and validation cohort. However, it is difficult to define a reasonable threshold probability for initiation of early treatment for AKI, and the threshold values may vary by setting and clinical experience of the attending physicians [31]. For AKI treatments, if we accept that all interventions are highly effective and carry fewer adverse effects, the acceptable threshold probability should be low, at 5–10% [33]. Unfortunately, at lower threshold probability, the use of AKI prediction did not significantly increase the NB over the strategy to treat all patients in the validation cohort. This could be explained by the higher prevalence of AKI in the validation cohort than in the derivation cohort. Thus, it might be safer to suggest that in a setting where AKI prevalence is high, the threshold probability of treatment initiation should be set even higher.

Several AKI prediction scores were developed and externally validated, especially in cardiac surgery [15,16,34,35] and orthopedic surgery [17]. For non-cardiothoracic surgery, the Simple Postoperative AKI Risk (SPARK) classification was developed in 2019 [36]. The SPARK classification was derived from a large cohort of 51,041 patients and was validated in a cohort of 39,764 patients from different centers in South Korea. The predictors included pre- and peri-operative determinants as follows: patients demographics (age and sex), comorbidity of diabetes mellitus, usage of renin-angiotensin-aldosterone-system blockage, laboratory investigations (baseline eGFR, hyponatremia, anemia, hypoalbuminemia, and albuminuria), and surgical information (expected surgical duration and emergency surgery status). The endpoints of interest were no-AKI, KDIGO AKI stage-I post-operatively, and critical AKI post-operatively (defined as KDIGO AKI stage II and III, dialysis in 90 days, or death). The SPARK score had a good discriminative ability (AuROC 0.80; 95% CI, 0.79–0.81) for forecasting post-operative AKI in the derivation cohort. However, after validation, a slight drop in discriminative ability to an acceptable range was reported (AuROC 0.72; 95% CI, 0.71–0.73).

There were several similarities and differences between the SPARK classification and our recently developed AKI prediction score. Firstly, although both scores were derived from surgical patients who underwent a major non-cardiothoracic operation, the SPARK population was not limited to critically ill patients admitted to the ICU post-operatively as ours was. As no baseline severity of the patients was reported in the SPARK classification development study, we assumed that our derivation cohort included a more severe domain of surgical patients. Secondly, common predictors from both scores were patients’ age and emergency surgery. Moreover, our prediction scores not only included pre- and peri-operative information, but also included post-operative information. Thirdly, although the KDIGO-AKI definition was used in both studies, the details of AKI prediction were differently described. In our previous study, AKI was defined during seven days after ICU admission, while post-operative AKI, AKI-related dialysis, and death were used in the SPARK study. Finally, the performance indices from both scoring systems seem to be similar in terms of generalizability. From the stage of development, both scores had a good discriminative performance; however, a trivial drop in a score performance to an acceptable range was identified during validation. This might be explained by a difference in case-mix, the prevalence of AKI in each cohort, the spectrum of disease severity, and distribution of predictors [31]. 

To the best of our knowledge, this was the first study to externally validate the predictive performance and clinical utility of the AKI prediction score for critically ill surgical patients who underwent a major non-cardiothoracic operation. As the score was developed from the THAI-SICU study, which was conducted four years earlier than the external validation dataset, this study should be considered a temporal validation [31]. The present study showed that our AKI prediction score, somehow, had generalizability with acceptable performance. Decision making for early aggressive interventions to prevent AKI guided by the score could be initiated before establishing definite AKI. The major strength of our study is in its prospective data collection. In addition, unlike most validation studies, we did not only report the discriminative ability, but we also reported the score calibration and the evaluation of clinical usefulness with DCA.

Our study had some limitations to be addressed. Firstly, a temporal dataset for external validation was retrospectively collected from a single center with a limited sample size and number of AKI events. Moreover, as the dataset used for validation was based on one of the THAI-SICU centers, the generalizability of the AKI prediction score to an entirely different group or population was still unknown. Further prospective studies should be conducted to evaluate the transportability of the AKI prediction score to other ethnic groups. Secondly, although the same definition for AKI was used in each cohort, differences in baseline clinical characteristics, the severity of the patients, the prevalence of AKI, and their outcomes between cohorts were identified. These differences clearly explained the decrease in the discriminative ability and score calibration. However, as the baseline characteristics of the patients in both cohorts were different, with higher severity in the validation cohort, it is likely that our study did not examine the reproducibility of the score but the transportability of the score to a more severe domain of surgical patients [37]. Finally, the categorization of continuous variables based on pre-defined fixed cut-off points of the original study might be another reason to explain the drop in the predictive performance of the score in the validation cohort. Re-categorization of cut-off points in a validation study that was much smaller, in terms of study size, might not be appropriate and may lead to excess optimism [25]. Thus, a totally fully independent validation study with a larger population should be conducted to recalibrate the AKI prediction score to find a more suitable cut-off point for continuous variables. Another alternative approach might be to apply flexible modeling of continuous predictors with fractional polynomial procedures or spline function and present the model as an equation, which can be embedded in a mobile application or a clinical decision support system.

## 5. Conclusions

In conclusion, the AKI prediction score provides acceptable discriminative ability and calibration upon external validation. Although the performance index decreased, the potential clinical utility of the score was shown from DCA. The AKI prediction score should be used as a clinical decision tool to help physicians decide whether an early aggressive intervention to prevent AKI should be initiated in each surgical patient admitted to the ICU following a major non-cardiothoracic operation. To further improve the ability of the score, an independent validation, score recalibration, or score updating in a larger population is encouraged. 

## Figures and Tables

**Table 1:** Baseline characteristics and clinical endpoints of the derivation cohort and the validation cohort.

Characteristics	Derivation (*n* = 3029)	Validation (*n* = 201)	Standardized Difference
Pre-operative			
Age (year, mean ± SD)	61.8 ± 16.7	62.5 ± 17.6	−0.042
Female, *n* (%)	1312 (43.4)	106 (52.7)	0.092
Body weight (kg, mean ± SD)	60.3 ± 15.9	61.6 ± 16.9	−0.081
Body mass index (kg/m^2^, mean ± SD)	23.3 ± 5.6	24.0 ± 5.9	−0.125
Pre-existing comorbidities			
Diabetes mellitus, *n* (%)	645 (21.3)	51 (25.4)	0.057
Hypertension, *n* (%)	1512 (49.9)	108 (53.7)	0.037
Cardiovascular diseases, *n* (%)	634 (20.9)	28 (13.9)	0.110
Respiratory diseases, *n* (%)	246 (8.1)	N/A	N/A
Chronic kidney disease, *n* (%)	267 (8.8)	38 (18.9)	0.242
Malignancy, *n* (%)	437 (14.4)	77 (38.3)	0.356
Others, *n* (%)	235 (7.8)	35 (17.4)	0.255
Peri-operative			
ASA classification, *n* (%)			0.259
I	193 (6.4)	12 (6.0)	
II–III	2470 (81.5)	179 (89.1)	
IV–V	366 (12.1)	10 (5.0)	
Emergency operation, *n* (%)	956 (31.6)	61 (30.4)	0.014
Operative sites, *n* (%)			
Neuro, head and neck surgery	344 (11.4)	22 (11.0)	0.010
Gastrointestinal surgery	1857 (61.3)	116 (42.3)	0.186
Orthopedics surgery	435 (14.4)	55 (27.4)	0.215
Others	494 (16.3)	39 (19.4)	0.053
Operative duration (min, mean ± SD)	280 ± 175	281 ± 147	−0.006
Peri-operative blood loss (mL, mean ± SD)	1026 ± 1787	728 ± 1077	0.170
Peri-operative fluid balance (mL, mean ± SD)	2112 ± 1852	1604 ± 1455	0.231
Peri-operative urine output (mL, mean ± SD)	505 ± 574	584 ± 537	−0.138
Post-operative (at ICU admission)			
APACHE - II score (mean ± SD)	10.6 ± 6.2	13.5 ± 5.8	−0.470
SOFA score (mean ± SD)	2.8 ± 3.0	3.9 ± 2.3	−0.371
SOFA non-renal score (mean ± SD)	2.2 ± 2.7	3.6 ± 2.2	−0.524
Sepsis	283 (9.3)	28 (13.9%)	0.117
Laboratory investigations			
Hemoglobin (gm/dL, mean ± SD)	10.7 ± 2.0	11.4 ± 1.9	−0.351
Albumin (gm/dL, mean ± SD)	2.78 ± 0.81	3.04 ± 0.75	−0.322
Blood glucose (mg/dL, mean ± SD)	166.8 ± 55.9	N/A	N/A
PaO_2_/FiO_2_ ratio (mean ± SD)	339 ± 129	N/A	N/A
Abnormal chest film, *n* (%)	436 (14.9)	N/A	N/A
Abnormal ECG, *n* (%)	678 (23.6)	N/A	N/A
Serum creatinine at ICU (mg/dL, mean ± SD)	1.19 ± 1.36	0.94 ± 0.59	0.189
Reference serum creatinine, *n* (%)			0.340
Renal dysfunction value	570 (18.8)	42 (20.9)	
MDRD recalculated value	943 (31.1)	34 (16.8)	
Lowest value during admission	1516 (50.1)	125 (62.2)	
Clinical endpoints			
AKI in 7 days of ICU admission, *n* (%)	296 (9.8)	37 (18.4)	0.198
ICU mortality, *n* (%)	159 (5.3)	10 (5.0)	0.014
Day-28 mortality, *n* (%)	251 (8.3)	12 (6.0)	0.080
ICU length of stay (day, mean ± SD)	3.2 ± 4.6	4.8 ± 5.8	−0.341
Hospital length of stay (day, mean ± SD)	20.9 ± 18.3	23.4 ± 19.3	−0.136
Average AKI prediction score			
AKI (mean ± SD)	8.5 ± 3.2	7.8 ± 3.5	0.216
Non-AKI (mean ± SD)	4.1 ± 2.9	4.9 ± 2.3	−0.279

Abbreviations: AKI, acute kidney injury; APACHE–II, Acute Physiology and Chronic Health Evaluation-II score; ASA, American Society of Anesthesiologist classification; ICU, intensive care unit; MDRD, the Modified of Diet in Renal Disease equation; PaO_2_/FiO_2_, ratio of arterial oxygen partial pressure to fractional inspired oxygen_;_ SD, standard deviation; SOFA, Sequential Organ Failure Assessment score.

**Table 2:** AKI prediction score performance for prediction of acute kidney injury in the derivation cohort and the validation cohort.

Score Performances	Derivation		Validation		Standardized Difference
	AKI/Total	% (95%CI)	AKI/Total	(95%CI)	
AuROC		86.9 (82.5–85.2)		74.5 (65.2–83.8)	0.245
Positive predictive value					
Low (0.0–2.5)	14/1212	1.2 (0.6–1.9)	1/36	2.8 (0.7–1.5)	0.181
Moderate (3.0–8.5)	142/1557	9.1 (7.7–10.7)	21/141	14.9 (9.5–21.9)	0.169
High (9.0–11.5)	95/264	36.0 (30.2–42.1)	9/17	52.9 (27.8–77.0)	0.167
Very high (12.0–16.5)	45/87	51.7 (40.8–2.6)	6/7	85.7 (42.1–99.6)	0.375
Total	296/3029	9.8 (8.7–10.9)	37/201	18.4 (13.3–24.5)	0.198

Abbreviations: AKI, acute kidney injury; AuROC, area under the receiver operating characteristic curve; CI, confidence interval.

**Table 3:** Decision curve analysis of the AKI prediction score in the derivation cohort and the validation cohort.

Threshold Probability for AKI (%)	Derivation			Validation		
	Treat All *	Net Benefit	Reduced Number of Overtreatment per 100 Patients	Treat All *	Net Benefit	Reduced Number of Overtreatment per 100 Patients
5	0.050	0.067	31.542	0.141	0.143	5.655
10	−0.003	0.051	48.516	0.093	0.094	1.818
15	−0.062	0.041	58.261	0.040	0.078	21.515
20	−0.128	0.031	63.420	−0.020	0.060	31.236
25	−0.203	0.023	67.712	−0.088	0.061	44.238
30	−0.289	0.016	71.199	−0.166	0.048	49.464
35	−0.388	0.009	73.710	−0.255	0.051	55.863
40	−0.504	0.003	76.080	−0.360	0.036	59.480
45	−0.641	−0.001	78.321	−0.483	0.035	63.381
50	−0.805	0.001	80.555	−0.632	0.030	66.169

Abbreviations: AKI, acute kidney injury. * Treat all refers to the treatment strategy to provide early aggressive intervention to prevent acute kidney injury in all patients.

## Data Availability

The datasets used and/or analyzed during the current study are available from the corresponding author on reasonable request.

## Supplementary Materials

The following are available online at https://www.mdpi.com/2227-9032/9/2/209/s1, Table S1. Multivariable risk predictors and their logit coefficients between derivation and external validation cohorts.
