# Supplementary material for: External Validation of the Acute Kidney Injury Risk Prediction Score for Critically Ill Surgical Patients Who Underwent Major Non-Cardiothoracic Surgery
Source: Healthcare (Basel). 2021 Feb 15;9(2):209. doi: 10.3390/healthcare9020209 (PMC7919279; doi:10.3390/healthcare9020209)
Supplement: Supplementary file 1 [file healthcare-09-00209-s001.pdf]

Table S1. Multivariable risk predictors and their logit coefficients between derivation and external validation cohorts.

Risk Factor		Derivation Cohort			External Validation Cohort		
	**Score**	**β**	**95%CI**	***p*-Value**	**β**	**95%CI**	***p*-Value**
**Patient demographic**							
Age < 65	0	Ref			Ref		
Age equal to 65 and above	1	0.477	0.198–0.755	0.001	1.356	0.426–2.287	0.004
**Perioperative data**							
Elective operation	0	Ref			Ref		
Emergency operation	2.5	0.899	0.595–1.203	< 0.001	0.838	−0.114–1.789	0.084
Perioperative blood loss							
< 1000 mL	0	Ref			Ref		
≥ 1000–2500 mL	1.5	0.535	0.161–0.909	0.005	1.735	0.680–2.789	0.001
> 2500 mL	2.5	1.043	0.647–1.440	< 0.001	1.056	−0.477–2.589	0.177
Perioperative urine output							
≥ 500 mL	0	Ref			Ref		
100–499 mL	1	0.384	0.033–0.735	0.032	−1.229	−2.188–0.269	0.012
< 100 mL	2.5	0.865	0.488–1.242	< 0.001	−0.324	−2.000–1.351	0.704
**Postoperative data (At ICU admission)**							
No sepsis	0	Ref			Ref		
Sepsis	3.0	1.223	0.886–1.560	< 0.001	2.509	0.578–4.440	0.011
SOFA non-renal *							
0–1	0	Ref			Ref		
2–5	2.5	0.990	0.626–1.353	< 0.001	0.861	−1.664–3.386	0.503
≥ 6	5.0	2.001	1.589–2.412	< 0.001	2.034	−0.588–4.655	0.128
Intercept		−4.631	−5.080–−4.182	< 0.001	−3.626	−6.332–−0.919	0.009

Abbreviations: β—logit coefficient; CI—confidence interval; ICU—intensive care unit; Ref—reference; SOFA—sequential organ failure assessment score.
